# A simple mathematical approach to the analysis of polypharmacology and polyspecificity data

**DOI:** 10.12688/f1000research.11517.1

**Published:** 2017-06-06

**Authors:** Gerry Maggiora, Vijay Gokhale

**Affiliations:** 1 BIO5 Institute, University of Arizona, 1657 East Helen Street, Tucson, AZ, 85719, USA

**Keywords:** drugs, drug targets, polypharmacology, polyspecificity, networks, edge-colored, bipartite networks, latent information

## Abstract

There many possible types of drug-target interactions, because there are a surprising number of ways in which drugs and their targets can associate with one another.  These relationships are expressed as polypharmacology and polyspecificity.  Polypharmacology is the capability of a given drug to exhibit activity with respect to multiple drug targets, which are not necessarily in the same activity class. Adverse drug reactions (‘side effects’) are its principal manifestation, but polypharmacology is also playing a role in the repositioning of existing drugs for new therapeutic indications.  Polyspecificity, on the other hand, is the capability of a given target to exhibit activity with respect to multiple, structurally dissimilar drugs.  That these concepts are closely related to one another is, surprisingly, not well known.  It will be shown in this work that they are, in fact, mathematically related to one another and are in essence ‘two sides of the same coin’.  Hence, information on polypharmacology provides equivalent information on polyspecificity, and *vice versa*.

Networks are playing an increasingly important role in biological research. Drug-target networks, in particular, are made up of drug nodes that are linked to specific target nodes if a given drug is active with respect to that target.  Such networks provide a graphic depiction of polypharmacology and polyspecificity.  However, by their very nature they can obscure information that may be useful in their interpretation and analysis.  This work will show how such latent information can be used to determine bounds for the degrees of polypharmacology and polyspecificity, and how to estimate other useful features associated with the lack of completeness of most drug-target datasets.

## Introduction

The study of drug-target interactions and their manifestation in polypharmacology and polyspecificity is playing a major role in the growing field of chemogenomics in particular, and in drug research in general. Polypharmacology describes the multiplicity of drug targets against which a given compound exhibits some form of biological activity ^1– 6^. A less appreciated characteristic of drug targets is their polyspecificity, namely the ability of multiple, structurally dissimilar drugs to exhibit biological activity against the same target.

The principal manifestation of polypharmacology is adverse drug reactions (‘side effects’), a phenomenon that has been recognized ever since the administration of the first drug ^7, 8^. In an interesting turnabout, side-effect similarity has recently been used to identify drug targets ^9^. A useful public data source called SIDER has also been developed; it links approximately 1000 drugs to nearly 1500 side effects ^10^. An emerging role of polypharmacology is in the repositioning of existing drugs for new therapeutic indications ^11^.

The term polyspecificity was primarily used to describe antibody recognition, and has been around for more than three decades ^12, 13^. It is only in the last few years, however, that it has been employed in the context of drug-target interactions. Consequently, there are fewer papers on this topic, and many of them deal with transporters and the efflux pumps that confer drug resistance ^14– 18^, which is hardly a broad sample of biological activity. This is somewhat surprising, given that the polyspecificity of drugs has not always been explicitly recognized as such. For a number of years, it has been manifest in many different forms in drug research, under the guise of multiple lead series ^19^, scaffold hopping ^20^, and pharmacophore-based structure-activity studies ^21^. All of these applications suggest that diverse structures may nevertheless exhibit biological activity with respect to the same target. This view is further supported by more recent evidence on the surprising prevalence of similarity cliffs ^22^, and indirectly by the enhanced effectiveness of group fusion in identifying new active compounds ^23^. These examples and the widespread occurrence of drug side effects suggest that some type of relationship might exist between polypharmacology and polyspecificity.

The alternative terminologies ‘drug promiscuity’ and ‘target promiscuity’ that are sometimes used instead of polypharmacology and polyspecificity, are slightly more general since they do not require the occurrence of biological activity, only that drugs and their targets interact ( *e.g.*, bind) in some specific fashion. Likewise, the term drug-target is sometimes replaced by the more general terms ligand-target or compound-target. However, the more popular although less general terms polypharmacology, polyspecificity, and drug-target will be used throughout the remainder of this work, with the caveat that their usage may sometimes be too narrow and may not always be strictly correct.

Recognition of the growing importance of polypharmacology in drug research and in biological research in general has resulted in the development of a number of drug-target databases ^24– 32^ summarized in Table 1. A cursory examination of these databases shows that most drugs, as well as many xenobiotics, apparently exhibit very high degrees of polypharmacology. However, the data in these databases needs to be considered with caution, because it may not be of uniform quality since many experimental methods or computational techniques of varying accuracy may have been used in its generation. This is further exacerbated by the fact that reproducing biological data can be difficult even when the same experimental method is used in different laboratories, or even in the same ones! The paper by Jasial ^33^ provides an interesting discussion that is relevant to this point.

**Table 1.** Sample of drug-target databases available over the Internet given by name, web address, and reference number in this work.

	Name	Web Address	Reference
1	DrugBank	www.drugbank.ca	24
2	STITCH	stitch.embl.de	25
3	WOMBAT	sunsetmolecular.com	26
4	PubChem BioAssay	ncbi.nlm.nih.gov/pcassay	27
5	BindingDB	bindingdb.org/bind/index.jsp	28
6	ChEMBL	ebi.ac.uk/chembl/target	29
7	canSAR	cansar.icr.ac.uk	30
8	PROMISCUOUS	bioinformatics.charite.de/promiscuous	31
9	MATADOR	omictools.com/matador-tool	32

To counter this issue database developers have established ‘reliability scores’ based on criteria of data quality, but there is no uniform procedure that is applied in all cases. Hence, drug-target datasets assembled with data obtained from multiple, diverse sources are unlikely to be of uniform quality. And this can give rise to significant uncertainties in the inferences that are drawn from analyses of such datasets.

By contrast, a number of more stringent evaluations have led to significantly reduced values for degrees of polypharmacology of many drugs ^33– 36^. But these values represent lower bounds to the true values, since the datasets from which these results are drawn are typically incomplete, an issue that is discussed further in this section. Additional study is certainly warranted in order to determine the true degree of polypharmacology for most drugs. As discussed in the following section, the multiplicity of ways that drugs can bind to a wide variety of different structural features in protein targets suggests the possibility that polypharmacology may be more prevalent than the most conservative view suggests. It does not, however, provide incontrovertible support for the extremely high degrees of polypharmacology implied by the data in many drug-target databases.

Data quality is not the only issue associated with drug-target datasets; another important concern is that of data completeness, as discussed in a recent paper by Mestres *et al.* ^37^. Data on all of the possible drug-target interactions within a given dataset of drugs and targets is generally unavailable, making a complete analysis of these interactions impossible. This issue is aggravated by the fact that almost all drug-target databases only report data on active compounds. The most complete datasets undoubtedly can be found in the laboratories of pharmaceutical companies, but since their data is proprietary it is of little value to researchers outside of these companies. The problem of data availability is also affected by biases that arise from the popularity of particular research areas such as GPCRs, ion channels, protein kinases, and proteases, which make up a significant portion of all targets in drug discovery research ^38^.

The crux of this paper is based on an analysis of the relationship between polypharmacology and polyspecificity, and it is demonstrated that they represent *mathematical duals* of one another. We describe (1) a rigorous mathematical relationship between polypharmacology and polyspecificity, based on a simple mathematical argument, and (2) an analysis of the latent information associated with drug-target interactions, described by edge-colored bipartite drug-target networks. The use of edge-colored networks provides the means for establishing bounds on the degrees of polypharmacology and polyspecificity. A simple example of a drug-target network is presented in order to clarify a number of the technical points raised in this paper. Currently, there is greater research focus on polypharmacology, since it has a seemingly more direct relationship to the pharmacological behavior of drugs. However, as far as we can determine, a definitive study rigorously linking polypharmacology and polyspecificity has yet to be published by other authors.

## Structural basis of drug-target interactions

It is important to recognize that polypharmacology and polyspecificity are purely phenomenological concepts. As such, they do not contain or require any specific structural information on the drugs or the targets they interact with. This is akin to classical chemical thermodynamics where, for example, the entropy, enthalpy, and free energy functions are purely phenomenological and do not in any way take account of the structural features of molecules ^39^. In the case of drug-target interactions, all that is needed is some measure of the degree of interaction, such as an activity, inhibition constant, or an IC _50_ value, all of which are phenomenological constants.

It has been generally assumed that in most instances of polypharmacology, the drug binding-site of one target or the domain within which it resides is in some fashion structurally related to the binding-site or domain of other targets that the drug interacts with ^40– 42^. A number of papers ^43– 46^ have taken a more high-resolution approach that focuses on individual groups within binding sites. The work from these laboratories has dramatically expanded the rather limited contemporary view of the structural requirements of drug-target interactions ^43– 46^. It counters the widely held, albeit changing, belief that if similar ligands bind to different proteins they must bind to structurally similar subsites in these proteins. The paper by Ehrt, *et al.* ^47^ provides an overview of this developing area of research.

Recent work from Shoichet’s group at UCSF is based on detailed structural studies of the binding of 59 different ligands in 116 complexes, where the binding of a given ligand involved pairs of proteins with different folds. In almost half of the protein pairs examined, a given ligand interacted with unrelated residues in the two proteins. Even in cases with similar binding-site environments, the ligands interacted with different residues. All of this shows that multiple patterns of residues and binding site environments are capable of interacting with highly structurally similar, even identical ligands. The investigators concluded that “ *There appears to be no single pattern-matching ‘code’ for identifying binding sites in unrelated proteins that bind identical ligands*”. This view is in line with what has been espoused by Mathews for protein-DNA interactions almost two decades earlier ^48^.

## Mathematical representations of drug-target interactions

### Drug-target relationships

Mathematically, drug-target interactions can be characterized as *binary relations*, R(D,T), that describe an association between a set of drugs

$$\text{D}=\left\{d_{1},d_{2},\ldots ,d_{n}\}\;(1\right)$$

and a set of drug targets

$$\text{T}=\left\{t_{1},t_{2},\ldots ,t_{m}\right\}\;.\;(2)$$

These relations are described by ordered-pairs of elements, ( *d __i__*, *t __j__*), formed by the Cartesian product of these two sets, D × T, *i.e.*

$$\left(d_{i},t_{j}\right)\in \text{R}\left(\text{D},\text{T}\right)\subseteq \text{D}\times \text{T}\;\text{for}\;\text{all}\;d_{i}\in \text{D}\;\text{and}\;t_{j}\in \text{T}.\;(3)$$

The meaning associated with ordered-pairs in a given relation depends on the nature of the relation. In this work we are interested in whether a drug is active with respect to a specific target. This is given by the characteristic function *r*( *d __i__*, *t __j__*) ∈ R associated with the relation, which satisfies

$$r\left(d_{i},t_{j}\right)=\{\begin{array}{l} 1\;\text{if}\;d_{i}\;\text{is}\;\text{active}\;\text{with}\;\text{respect}\;\text{to}\;t_{j}\\ 0\;\text{if}\;d_{i}\;\text{is}\;\text{inactive}\;\text{with}\;\text{respect}\;\text{to}\;t_{j} \end{array},\;(4)$$

where the activity values are equal to or greater than a threshold value that typically lies in the range of 1μM -10 μM. The elements *r*( *d __i__*, *t __j__*) are generally collected into a *n* × *m* dimensional matrix,

$$\text{R}=\left[\begin{array}{cccc} r\left(d_{1},t_{1}\right) & r\left(d_{1},t_{2}\right) & \cdots & r\left(d_{1},t_{m}\right)\\ r\left(d_{2},t_{1}\right) & r\left(d_{2},t_{2}\right) & \cdots & r\left(d_{2},t_{m}\right)\\ \vdots & \vdots & \ddots & \vdots \\ r\left(d_{n},t_{1}\right) & r\left(d_{n},t_{2}\right) & \cdots & r\left(d_{n},t_{m}\right) \end{array}\right].\;(5)$$

Now consider the *transpose* of the relation, R(D,T)′ = R(T,D). This changes the order of the elements in the ordered-pairs, *i.e.*

$$\left(d_{i},t_{j})\in \text{R}\left(\text{D},\text{T}\right)\to (t_{j},d_{i})\in \text{R}{\left(\text{D},\text{T}\right)}^{\prime }\;(6\right)$$

Nothing has fundamentally changed, except the arrangement of the elements of the relation; their values remain the same

$${\text{R}}^{\prime }=\left[\begin{array}{cccc} r\left(t_{1},d_{1}\right) & r\left(t_{1},d_{2}\right) & \cdots & r\left(t_{1},d_{n}\right)\\ r\left(t_{2},d_{1}\right) & r\left(t_{2},d_{2}\right) & \cdots & r\left(t_{2},d_{n}\right)\\ \vdots & \vdots & \ddots & \vdots \\ r\left(t_{m},d_{1}\right) & r\left(t_{m},d_{2}\right) & \cdots & r\left(t_{m},d_{n}\right) \end{array}\right].\;(7)$$

Now the relation can be viewed from the ‘target perspective’. This clearly shows that however the values of the elements of R(D,T) or R(D,T)′ are obtained, *i.e.* from the drug perspective *r*( *d __i__*, *t __j__*), which is associated with polypharmacology, or from the target perspective *r*( *t __j__*, *d __i__*), which is associated with polyspecificity, the two views are completely comparable. The above argument is the basis for showing that polypharmacology and polyspecificity are mathematical duals of one another.

In order to simplify and clarify all subsequent discussion, the following three categories of relations associated with ordered drug-target pairs are defined:

(1) ‘ *active*’, which includes all drug-target pairs whose activity has been experimentally measured or computationally estimated to meet or exceed the designated activity threshold value;

(2) ‘ *inactive*’, which includes all drug-target pairs whose activity value has been experimentally measured or computationally estimated to fall below the designated activity threshold value; and

(3) ‘ *unknown*’, which includes all drug-target pairs whose activities have neither been measured experimentally nor estimated computationally.

The following simple, illustrative example shows that the 8 × 4 dimensional drug-target interaction matrix and its transpose, the 4 × 8 target-drug interaction matrix, contain entirely equivalent information – only the ‘viewpoint’ has changed:

$$\begin{array}{cc} {\text{R}}_{\text{+}}=\left[\begin{array}{cccc} 1 & 1 & 0 & 1\\ 0 & 1 & 1 & 0\\ 1 & 0 & 0 & 0\\ 1 & 1 & 1 & 0\\ 0 & 1 & 0 & 1\\ 1 & 0 & 0 & 1\\ 1 & 1 & 1 & 1\\ 0 & 0 & 1 & 1 \end{array}\right], & {\text{R}}_{\text{+}}^{\prime }=\left[\begin{array}{cccccccc} 1 & 0 & 1 & 1 & 0 & 1 & 1 & 0\\ 1 & 1 & 0 & 1 & 1 & 0 & 1 & 0\\ 0 & 1 & 0 & 1 & 0 & 0 & 1 & 1\\ 1 & 0 & 0 & 0 & 1 & 1 & 1 & 1 \end{array}\right] \end{array}.\;(8)$$

In **R**_+_, the rows correspond to drugs and the columns to targets, while in ${\text{R}}_{+}^{\prime }$ the rows correspond to targets and the columns to drugs. The positive subscript indicates that the matrix represents active drug-target pairs.

### Bipartite networks

It may also be desirable to represent the information in Equations (5), (7), and (8) as a network ^49, 50^, since a considerable amount of the data on biological interactions is presented in the literature as networks. When the entities that are being compared belong to different sets, for example drugs and targets, a bipartite network such as that given in Equation (9) is commonly used:

N=〈D∪T,E〉.(9)

These networks are comprised of sets of drug and target nodes, D and T, that are non-overlapping, i.e. D ∩ T = ∅. Edges only link nodes between D and T; there are no edges linking pairs of nodes within either D or T. Thus, the edge set can be defined as

E={e(di,tj)|if(di,tj)isan'active'drug-targetpair}(10)

In networks, pairs of nodes directly linked by edges are said to be adjacent and constitute the elements of the ( *n* + *m*) × ( *n* + *m*) dimensional adjacency matrix:

$${\tilde{A}}_{\left(n+m)\times (n+m\right)}=\left[\begin{array}{ll} 0_{n\times n} & A_{n\times m}\\ {A^{\prime }}_{m\times n} & 0_{m\times m} \end{array}\right]\;,\;(11)$$

where

$$A=\left[\begin{array}{cccc} a(d_{1},t_{1}) & a(d_{1},t_{2}) & \cdots & a(d_{1},t_{m})\\ a(d_{2},t_{1}) & a(d_{2},t_{2}) & \cdots & a(d_{2},t_{m})\\ \vdots & \vdots & \ddots & \vdots \\ a(d_{n},t_{1}) & a(d_{n},t_{2}) & \cdots & a(d_{n},t_{m}) \end{array}\right]\;,\;(12)$$

is called a biadjacency ^51^ or incidence matrix ^49^, although the latter usage is not strictly correct. The elements of **A** indicate which nodes of D are adjacent (i.e. linked or connected) to those of T. This provides what could be called drug-based view of the network. Since **A**′ is the transpose of **A**, its elements now indicate which nodes of T are adjacent to those of D ( *Cf*. Equation (6)). This can be said to provide a target-based view of the network. Because the same information is contained in both matrices, the corresponding network has no directionality and is thus an undirected network. Moreover, the network topology is independent of which representation is used.

While not technically correct, for simplicity in this work **A** will be termed the adjacency matrix of , since it contains all of the information in . The zero valued submatrices in $\tilde{A}$ show that there are no links among nodes within D or among those within T. *Since the elements of* **A** *are in one-to-one correspondence with the elements of* **R**, *the two matrices are isomorphic*. Hence, **R** and **A**, and by implication , contain essentially the same information.

**Figure 1.**
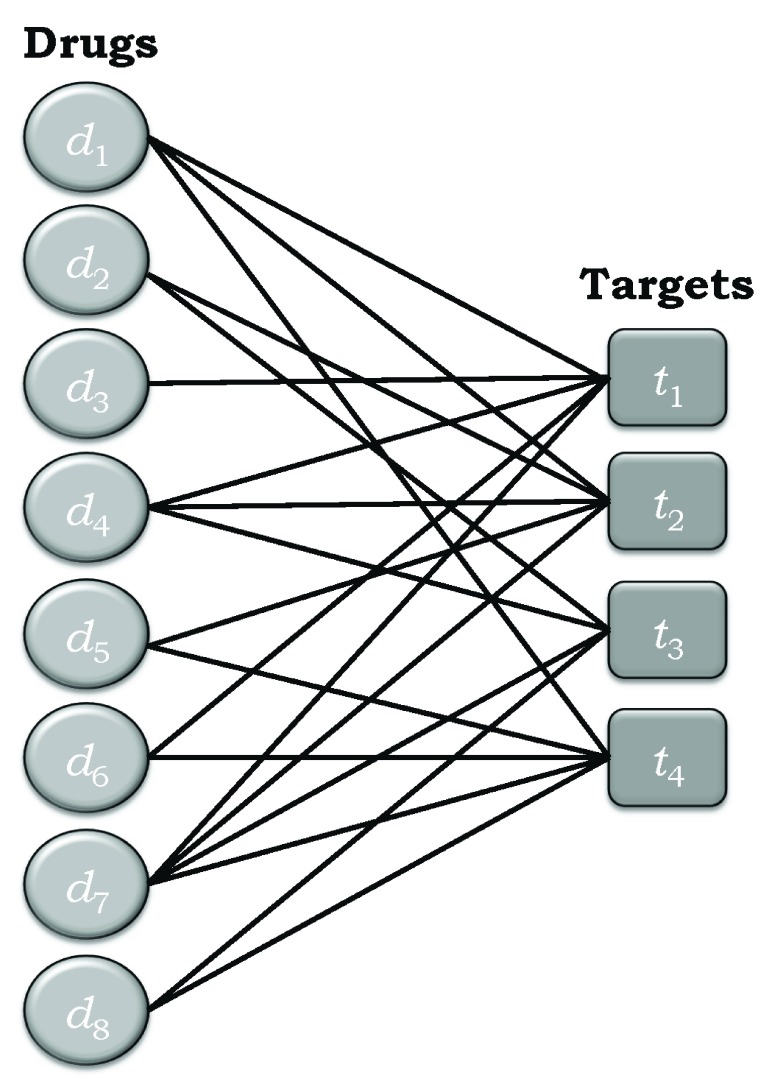
Simple example of a bipartite drug-target network made up of eight drugs and four targets.

Figure 1 depicts the bipartite network corresponding to the drug-target interaction matrix **R**_+_ given Equation (8). From the discussion of the general relationship of **R** and **A** in the previous paragraphs it follows that

$$A_{+}=\left[\begin{array}{cccc} 1 & 1 & 0 & 1\\ 0 & 1 & 1 & 0\\ 1 & 0 & 0 & 0\\ 1 & 1 & 1 & 0\\ 0 & 1 & 0 & 1\\ 1 & 0 & 0 & 1\\ 1 & 1 & 1 & 1\\ 0 & 0 & 1 & 1 \end{array}\right]=R_{+}\;.\;(13)$$

Note that ${\text{R}}_{+}^{\prime }$ and ${\text{A}}_{+}^{\prime }$ interchange the positions of the nodes of the corresponding network, so that the target nodes now lie on the left hand side of the network diagram and the target nodes lie on the right hand side. This changes nothing, since the topology of the network is the same in both cases.

## Drug-target networks

### Network data

Yildrim, *et al.* ^52^ provided the earliest example of drug-target networks. Vogt and Mestres ^53^ have also discussed a number of issues associated with such networks including, as mentioned earlier, the issue of data completeness ^37^. Other related databases have also been developed such as those based on drug-side effects ^10^ and gene-disease networks ^54^.

While it is true that drug-target networks provide dramatic views of the complex interrelationships amongst drugs and their putative targets, they are difficult to interpret when the number of drug-target pairs becomes too large, as is demonstrated by several of the figures depicted in references 52 and 53. In those cases networks merely provide a visual sense of drug-target relationships and their overall complexity.

Because of this, such networks are rarely used directly to draw detailed inferences. Rather, as the information contained within them is available in various matrices such as the adjacency matrices shown in Equations (11) – (13), it can be analyzed by algebraic procedures, some of which are described in this work. However, even the matrix algebraic approach becomes limiting for the adjacency matrices of large drug-target systems, which are quite sparse. In such cases, normal matrix-algebraic procedures become very inefficient. Storing the limited amount of data in such large sparse matrices is also very wasteful. This necessitates the development of efficient data structures and algorithmic procedures that facilitate the management and analysis of large drug-target datasets ^55^. The fact that so many large networks such as the Internet have been analyzed has led to the development of highly efficient algorithms that are more than capable of handling the size problems typically encountered with drug-target networks. The last part of the book by Newman ^49^ describes a number of these algorithms. They are not employed here, since the goal of the current work is the development of an understanding of some of the overlooked characteristics of drug-target network data and their analysis. Consequently, a very simple example is used as a basis for describing the underlying principles.

Many databases have been developed in order to provide a more unified source of experimental and computational data on drug-target interactions. Table 1 provides a summary of some useful drug-target databases. References to the various experimental methods used can best be found in the databases themselves. Because of the size and complexity of the chemogenomic space, computational methods have begun to play a larger role in determining drug-target interactions. A sample of some of the many computational techniques is given in the following references ^6, 56– 59^.

### Polypharmacology and polyspecificity

The work described here is based on a phenomenological model of interactions between a set of drugs and a corresponding set of targets. Thus, as noted earlier, there is no requirement for any information on the molecular structure of the drugs, their targets, or any details on the nature of their inter- molecular interactions.

The degree of a given drug node is equal to the number of edges connected to that node, which is equivalent to the degree of polypharmacology of the drug associated with that node. The degree of a given target node is equivalent its degree of polyspecificity. It should be clear from Figure 1 that knowing the polypharmacology associated with the drug nodes is tantamount to knowing the degree of polyspecificity of the target nodes, and *vice versa*.

That this is the case can also be seen from the relational matrix, **R**_+_, given by Equation (8) or from the adjacency matrix, **A**_+_, given by Equation (13). In both instances, the rows represent drugs and the columns targets. Rows can be thought of as binary vectors associated with each of the drugs whose components are the targets the drugs can potentially interact with; correspondingly, columns can be thought of as binary vectors associated with each of the targets whose components are the drugs they can potentially interact with. Thus, all of the information on the degrees of polypharmacology and polyspecificity are contained in **R**_+_ and **A**_+_. Polypharmacology data, polyspecificity data, or some combination of the two can be used to ‘fill in’ the elements of **R**_+_ and **A**_+_. The degrees of polypharmacology and polyspecificity can then be computed by the expressions given in Equation (14) where the row and column sums correspond to the usual nodal degrees of the drug and target nodes, *k̂*_+_( *d __i__*) and *k̂*_+_( *t __j__*), which are equivalent to their corresponding degrees of polypharmacology and polyspecificity, π̂ __PP__( *d __i__*) and π̂ __PS__( *t __j__*), *i.e.*

$$\begin{array}{l} {\hat{k}}_{+}(d_{i})\equiv {\hat{\pi }}_{\text{PP}}(d_{i})=\sum_{t_{j}\in \text{T}}a_{+}(d_{i},t_{j}),\;\text{for}\;\text{all}\;d_{i}\in \text{D}\\ {\hat{k}}_{+}(t_{j})\equiv {\hat{\pi }}_{\text{PS}}(t_{j})=\sum_{d\in \text{D}}a_{+}(d_{i},t_{j}),\;\text{for}\;\text{all}\;t_{j}\in \text{T} \end{array}\;,\;(14)$$

where the *a*_+_( *d __i__*, *t __j__*) are elements of **A**_+_. Note that use of the caret or circumflex symbol "∧" follows customary statistical usage and indicates that these values are estimates. This will be used consistently throughout this manuscript to indicate parameters estimated from data in the corresponding relational or adjacency matrices. As noted earlier, the adjacency or relational matrices contain all of the information needed to determine the degrees of polypharmacology and polyspecificity, in this case for the set of eight drugs and four targets, regardless of how the data are created. Having information about one of them automatically provides information on the other, since *r*( *d __i__*, *t __j__*) = *r*( *t __j__*, *d __i__*) and *a*( *d __i__*, *t __j__*) = *a*( *t __j__*, *d __i__*).

Table 2 summarizes the degrees of polypharmacology and polyspecificity for the sets of drugs and targets in the example depicted in Figure 1, and represented by the adjacency matrix in Equation (13). But there is more that needs to be considered.

**Table 2.** Active drug-target interactions. The rows correspond to drugs and the columns to targets. The far right hand column gives values for the degree of polypharmacology, while the bottom most row gives values for the degree of polyspecificity. The binary values at the center of the table show whether a given drug-target pair is active (1) or inactive (0) or of unknown activity (0).

	*t*_1_	*t*_2_	*t*_3_	*t*_4_	Polypharmacology
***d*_1_**	1	1	0	1	3
***d*_2_**	0	1	1	0	2
***d*_3_**	1	0	0	0	1
***d*_4_**	1	1	1	0	3
***d*_5_**	0	1	0	1	2
***d*_6_**	1	0	0	1	2
***d*_7_**	1	1	1	1	4
***d*_8_**	0	0	1	1	2
**Polyspecificity**	5	5	4	5	19

### Limitations of network representations

The network representation of drug-target interactions effectively captures the information associated with active drug-target pairs, but in many instances it does not capture comparable information on inactive drug-target pairs or pairs whose activities have not been evaluated experimentally or computationally. This can lead to considerable uncertainty in the dataset and can be a latent source of error in the determination of degrees of polypharmacology and polyspecificity. The situation is exacerbated by the fact that most drug-target databases do not report data on drugs that are inactive, even if such data exists. In those cases, the drug-target pair must be assumed to belong to the category of pairs with *unknown* activity. How this affects the analysis of drug-target interactions is described in the sections that follow.

It is quite likely that within larger datasets, the activity of many of the drug-target pairs has not been evaluated experimentally or computationally. Since some of these may nevertheless be active, it follows that the degrees of polypharmacology and polyspecificity are typically underestimated and hence only provide approximate lower bounds to the true values. They are not true lower bounds because the data used for their determination are not always entirely consistent or accurate. Hence their reliability may be questionable.

Even though the number of drug-target pairs in the inactive and unknown categories is small in the example given here, in reality the number can be substantial and generally exceeds the number of active drug-target pairs. This makes total sense given that the number of active compounds in large corporate databases is generally only a few percent of the total number of compounds in their database. Thus, the problem now becomes how to obtain data on drugs in a dataset that are known to be inactive. As mention earlier, this is a significant problem for two reasons. First, activity data in corporate databases, where such information is likely to exist, is generally unavailable to the general research community. Second, most databases accessible by the non-industrial research community either do not report or report very little data on inactive drugs. Because of this, it is difficult to determine the contributions of drugs to the inactive category, which directly affects our knowledge of drugs in the category of unknown activity status. As will be seen in a forthcoming section, this impacts the size of the bounds to the degrees of polypharmacology and polyspecificity. Thus, while data on inactive drug-target pairs does not provide information that is useful for identifying drug targets, its availability reduces the size of the category of drugs of unknown activity, which improves the bounds on the degrees of polypharmacology and polyspecificity. The details of this argument are presented in a forthcoming section and are exemplified by the expression given in Equation (22).

More importantly, in many cases the number of possible drug-target pairs whose activity status is unknown may be significant. If they were experimentally or computationally determined, at least some of these might have activity values that meet or exceed the desired activity threshold. Not including these data will result in a less reliable estimation of the degrees of polypharmacology and polyspecificity. It may also suggest that the observed drug-target interactions involve a more limited region of target space than is actually the case. All of these issues raise questions as to how such data can be effectively incorporated into an analysis of drug-target interactions. One way to address this issue is by extending the current networks to include the class of edge-colored bipartite networks.

### Edge-colored bipartite networks

**Figure 2.**
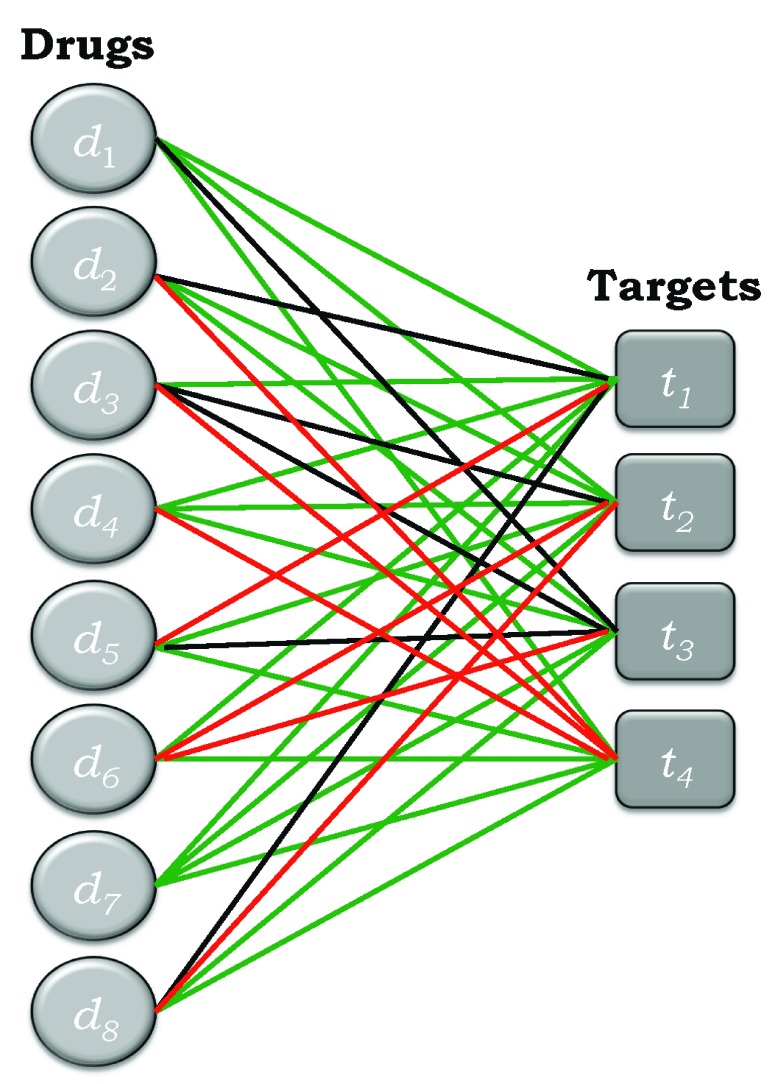
Example of the network in Figure 1 represented as an edge-colored network, where the green edges correspond to active drug-target pairs, the red edges to inactive drug-target pairs, and the black edges to drug-target pairs of unknown activity status.

**Figure 3.**
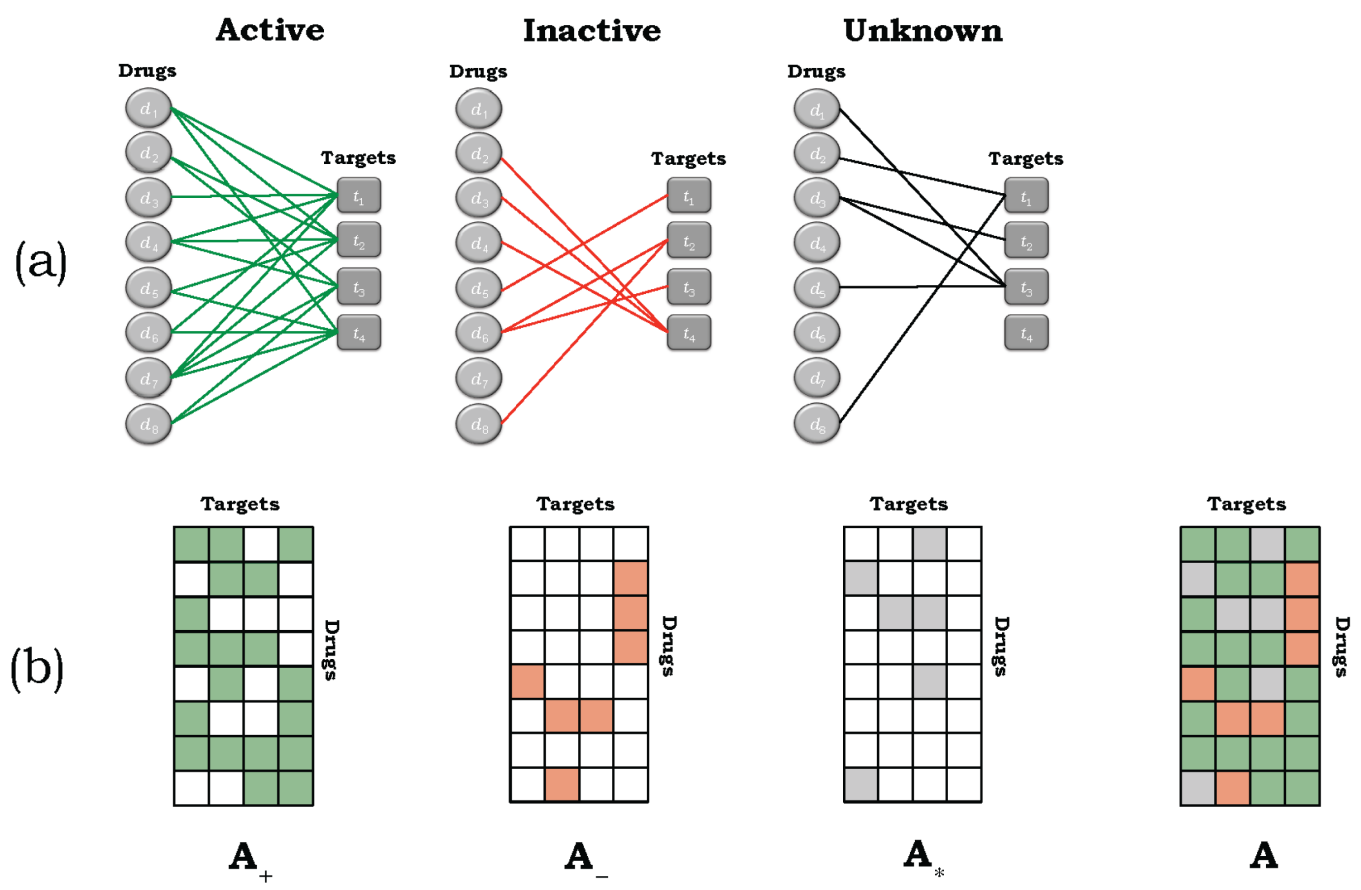
( **a**) Decomposition of the bipartite, edge-colored network depicted in Figure 2 into its three component subnetworks, namely drug-target pairs that are active, inactive, and of unknown activity status. ( **b**) The adjacency matrices corresponding to the bipartite, edge-colored subnetworks given in ( **a**). The colored cells correspond to a value of unity and the uncolored cells to zero values.

An edge-colored bipartite network is depicted in Figure 2 for the simple example shown in Figure 1. Edges corresponding to active drug-target pairs are colored green, those corresponding to inactive pairs are colored red, and those corresponding to pairs of unknown activity are colored black. Thus, all of the possibilities are now incorporated into a single edge-colored network. Figure 3a represents a separation of this network into its three components, corresponding to active (+), inactive (−), and unknown (*) bipartite subnetworks. Figure 3b depicts their respective adjacency matrices, **A**_+_, **A**_-_, and A_*_, where the colored squares correspond to matrix elements with value ‘1’ and the uncolored squares correspond to matrix elements with value ‘0’. An examination of Figure 3b shows that the matrix elements of **A**, **A**_+_, **A**_-_, and A_*_ satisfy

$$\begin{array}{l} a(d_{i},t_{j})=a_{+}(d_{i},t_{j})+a_{-}(d_{i},t_{j})+a_{*}(d_{i},t_{j})=1\;\\ \;\;\text{for}\;\text{all}\;d_{i}\in \text{D}\;\text{and}\;t_{j}\in \text{T} \end{array}\;(15)$$

The elements of the three matrices cover all possible drug-target interactions and are non-overlapping. Thus, they represent a partition of the matrix elements of **A**, all of whose elements have value unity.

Because of this, it is possible to determine the degrees of nodes for each of the subnetworks independently. Thus, the row and column sums for the three colored networks associated with **A**_+_, **A**_-_, and A_*_, are given, respectively, by

$$\begin{array}{l} {\hat{k}}_{\eta }(d_{i})=\sum_{y_{j}\in \text{Y}}a_{\eta }(d_{i},t_{j})\;\text{for}\;\text{all}\;d_{i}\in \text{X}\\ {\hat{k}}_{\eta }(t_{j})=\sum_{x_{i}\in \text{X}}a_{\eta }(d_{i},t_{j})\;\text{for}\;\text{all}\;t_{j}\in \text{Y} \end{array}\;(16)$$

where η ≜ +, –, *. Equation (14) shows the equivalences *k̂*_+_( *d __i__*) ≡ π̂ __PP__( *d __i__*) and *k̂*_+_( *t __j__*) ≡ π̂ __PS__( *t __j__*). As is discussed in detail in forthcoming sections, the terms k̂_*_( *d __i__*) and k̂_*_( *t __j__*) are equivalent to error terms that provide uncertainty measures with respect to the degrees of polypharmacology and polyspecificity. In order to emphasize this property and to make their association with π̂ __PP__( *d __i__*) and π̂ __PS__( *t __j__*) clear, the following equivalences are defined: k̂_*_( *d __i__*) ≡ ε̂ __PP__( *d __i__*) and k̂_*_( *t __j__*) ≡ ε̂ __PS__( *t __j__*) for all *d __i__* ∈ D and *t __j__* ∈ T.

The results for the simple example depicted in Figure 1– Figure 3 are collected in Table 3 and Table 4. In Table 3, *k̂*_-_( *d __i__*) corresponds to the right hand column designated ‘Row-Sum’, and *k̂*_-_( *t __j__*) corresponds to the bottom row designated ‘Col-Sum’, and similarly for ε̂ __PP__( *d __i__*) and ε̂ __PS__( *t _j_*), respectively, in Table 4. These latter quantities associated with the drug-target pairs of unknown activity are important since they contain information, albeit latent information, that bears on the degrees of polypharmacology and polyspecificity for any drug-target dataset. As noted earlier, some of the drugs known to be inactive may nonetheless fall in the category of drugs of unknown activity, because inactivity data is not generally incorporated into many of the widely available drug-target databases. Moreover, the terms associated with inactive drug-target pairs *k*_-_( *d __i__*) and *k*_-_( *t __j__*) provide useful information since they eliminate the possibility of being considered as active pairs. They also have an effect on the sizes of ε̂ __PP__( *d __i__*) and ε̂ __PS__( *t _j_*), as discussed in a forthcoming section.

**Table 3.** Inactive drug-target interactions. The rows correspond to drugs and the columns to targets. The far right hand column gives values for the row sums (‘Row-Sum’), while the bottom most row gives values for the corresponding column sums (‘Col-Sum’). The binary values at the center of the table show whether a given drug-target pair is inactive (1) or active (0) or of unknown activity (0).

	*t*_1_	*t*_2_	*t*_3_	*t*_4_	Row-Sum
***d*_1_**	0	0	0	0	0
***d*_2_**	0	0	0	1	1
***d*_3_**	0	0	0	1	1
***d*_4_**	0	0	0	1	1
***d*_5_**	1	0	0	0	1
***d*_6_**	0	1	1	0	2
***d*_7_**	0	0	0	0	0
***d*_8_**	0	1	0	0	1
**Col-Sum**	1	2	1	3	7

**Table 4.** Unknown drug-target interactions. The rows correspond to drugs and the columns to targets. The far right hand column gives values for the row sums (‘Row-Sum’), while the bottom most row gives values for the corresponding column sums (‘Col-Sum’). The binary values at the center of the table show whether a given drug-target pair is of unknown activity (1) or active (0) or inactive (0).

	*t*_1_	*t*_2_	*t*_3_	*t*_4_	Row-Sum
***d*_1_**	0	0	1	0	1
***d*_2_**	1	0	0	0	1
***d*_3_**	0	1	1	0	2
***d*_4_**	0	0	0	0	0
***d*_5_**	0	0	1	0	1
***d*_6_**	0	0	0	0	0
***d*_7_**	0	0	0	0	0
***d*_8_**	1	0	0	0	1
**Col-Sum**	2	1	3	0	6

**Figure 4.**
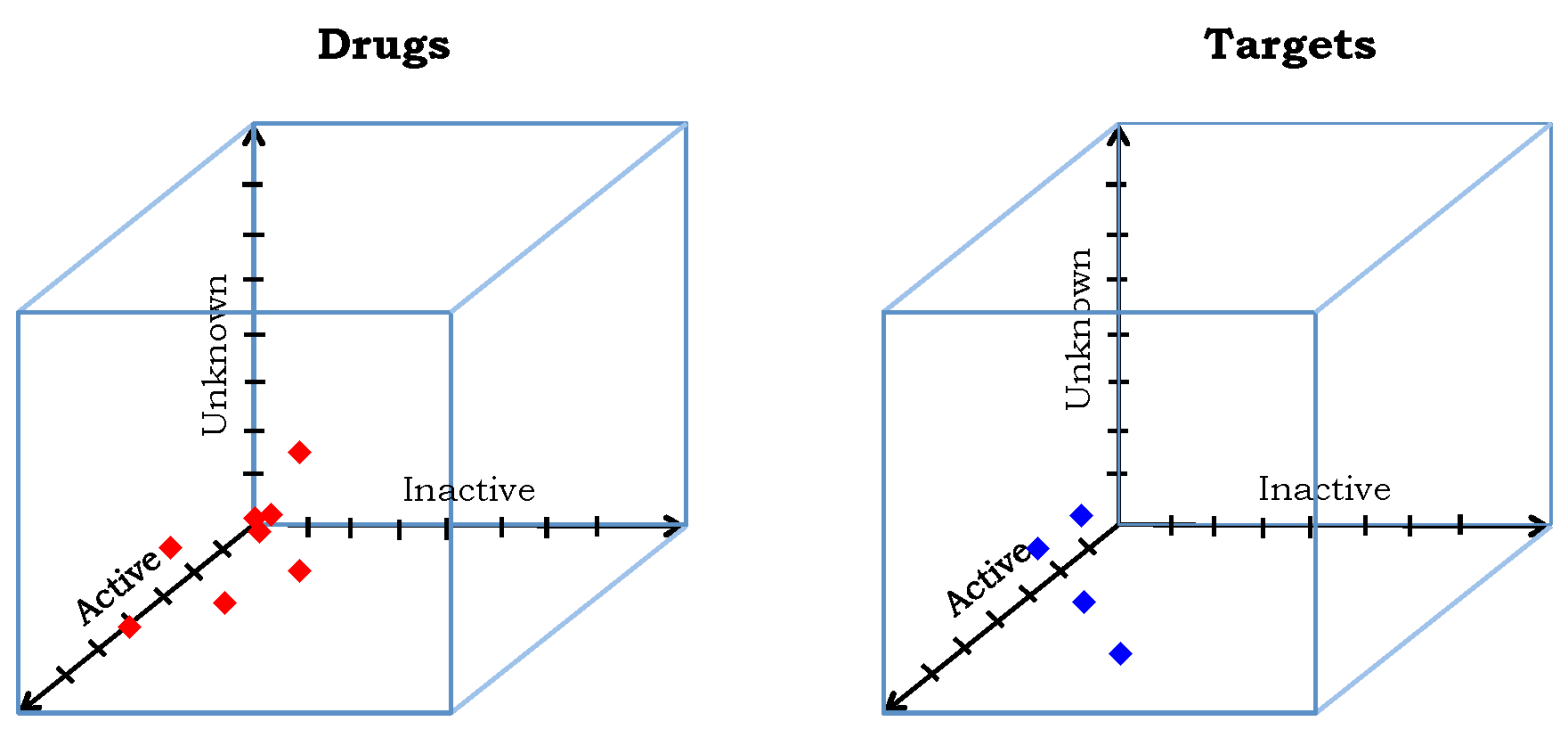
( **a**) Three-dimensional plots of the information in Table 2– Table 4 for drugs. ( **b**) Three-dimensional plots of the information in Table 2– Table 4 for targets.

The information in Table 2– Table 4 can be represented as three-dimensional Euclidean vectors

$$\begin{array}{l} k_{\text{PP}}(d_{i})=\left({\hat{\pi }}_{\text{PP}}(d_{i}),{\hat{k}}_{-}(d_{i}),{\hat{\varepsilon }}_{\text{PP}}(d_{i})\right)\\ k_{\text{PS}}(t_{j})=\left({\hat{\pi }}_{\text{PP}}(t_{j}),{\hat{k}}_{-}(t_{j}),{\hat{\varepsilon }}_{\text{PP}}(t_{j})\right) \end{array}\;(17)$$

that can be plotted in three dimensions as depicted in Figure 4. Although not examined in this work, Euclidean vectors also allow computation of inter-vector distances and cosine-based similarities ^23^, either of which can be used to cluster the data points by a variety of well-known methods ^60^.

In the case where the activities of all of the drug-target pairs have been measured, ideally the points will lie entirely within the ‘Active-Inactive’ plane. In general, the information provided exceeds that of typical bipartite drug-target networks, because of the explicit inclusion of data on drug-target pairs of inactive and unknown activity.

## Measures of data completeness

### Global measures

A global measure of data completeness that accounts for experimentally determined or computationally estimated activities of drug-target pairs is given by

$${\hat{C}}_{\text{DT}}=\frac{{\hat{\mu }}_{+}+{\hat{\mu }}_{-}}{{\hat{\mu }}_{+}+{\hat{\mu }}_{-}+{\hat{\mu }}_{*}}\;(18)$$

where μ̂ _+_ is an estimate of the total number of experimentally or computationally determined active pairs, μ̂ _−_ is an estimate of the total number of experimentally or computationally determined inactive pairs and μ̂ _*_ is an estimate of the total number of pairs of unknown activity status. Thus,

$${\hat{\mu }}_{\eta }=\sum_{d_{i}\in \text{D}}\sum_{t_{j}\in \text{T}}a_{\eta }(d_{i},t_{j})\;(19)$$

where η ≜ +, −, *. The denominator of Equation (18) is a known constant because it is equal to the total number of possible drug-target pairs in the dataset, | D × T | = *n*· *m*. Hence, there are only two degrees of freedom for the estimated quantities, and the value of μ̂ _*_ is specified directly if the values of μ̂ _+_ and μ̂ _−_ are known.

In the example given in Figure 2 and Figure 3 and Equation (8) and Equation (13), and μ̂ _+_ = 19, μ̂ _−_ = 7, μ̂ _*_ = 6. Thus,

$$C_{\text{DT}}=\left(19+7\right)/\left(19+7+6\right)=26/32=0.813,\;(20)$$

which satisfies 0 ≤ *C*__DT__ ≤ 1. Obviously, the closer *C*__DT__ is to unity, the more accurate the estimates of polypharmacology and polyspecificity will be, but it provides no information on the degrees of polypharmacology and polyspecificity associated with individual drugs or targets.

### Local measures

In many instances, it is desirable to have local measures that are associated with individual drug or target nodes. One possible local measure is related to the nodal degrees of bipartite subnetworks associated with drug-target pairs of unknown activity status, ε̂ __PP__( *d __i__*) and ε̂ __PS__( *t _i_*), which can be viewed as measures of error or uncertainty. Fractional measures could also be defined by dividing each of them by | T | and | D |, respectively, but this will not be done here.

In order to develop these measures, the nodal degrees are combined with respect to all three types of relations given by Equation (16) for each of the nodes *d __i__* ∈ D and *t _j_* ∈ T. Combining and simplifying terms using Equation (15) yields

$$\begin{array}{l} {\hat{\pi }}_{\text{pp}}(d_{i})+{\hat{k}}_{-}(d_{i})+{\hat{\varepsilon }}_{\text{pp}}(d_{i})=|\text{T}|\;\text{for}\;\text{all}\;d_{i}\in \text{D}\\ {\hat{\pi }}_{\text{PS}}(t_{j})+{\hat{k}}_{-}(t_{j})+{\hat{\varepsilon }}_{\text{PS}}(t_{j})=|\text{D}|\;\text{for}\;\text{all}\;t_{j}\in \text{T} \end{array}.\;(21)$$

As was the case for the variables in the denominator of Equation (18), the sum of terms in either expression in Equation (21) is equal to a constant, and hence there are two degrees of freedom. Once values for the first two terms in either expression of Equation (21) are obtained by appropriately summing the experimentally or computationally determined elements of their corresponding adjacency matrices **A**_+_ and **A**_−_, the values of the remaining error terms, ε̂ __PP__( *d __i__*) and ε̂ __PS__( *t _i_*), are automatically specified. Nevertheless, uncertainties in these terms remain because it is not known which of their elements, a__*__( *d _i_, t _j_*), correspond to active drug-target pairs, *i.e.* which have a value of unity, and which do not.

Knowing that the values of *k*_−_( *d __i__*) and *k*_−_( *t __j__*) are useful is seen by rearranging Equation (21)

$$\begin{array}{l} {\hat{\varepsilon }}_{\text{PP}}(d_{i})=\left|\text{T}\right|-{\hat{\pi }}_{\text{PP}}(d_{i})-k_{-}(d_{i})\\ {\hat{\varepsilon }}_{\text{PS}}(t_{j})=\left|\text{D}\right|-{\hat{\pi }}_{\text{PS}}(t_{j})-k_{-}(t_{j}) \end{array}.\;(22)$$

The following example illustrates this point. Consider a specific drug, say *d _p_*, with unknown activity with respect to a subset of two of the targets under study; hence, ε̂ __PP__( *d _p_*) = 2. Now experimentally or computationally determine the activity of the drug with respect one of the targets, say *t _q_*. The drug will either be active or inactive. Regardless of which, it will diminish the size of ε̂ __PP__( *d _p_*) = 2 and, as will be seen in the following section, will tighten the bounds on π̂ __PP__( *d _p_*). Hence, even though the compound has no particular value as a drug for that target, knowing that it is inactive improves the estimate of its degree of polypharmacology with respect to the entire set of targets under study. This affords a clear example of the usefulness of information on the inactivity of drugs towards specific targets. An exactly analogous argument can be made regarding targets, although the details will not be given here.

## Bounds for the degrees of polypharmacology and polyspecificity

Bounds to the values of π̂ __PP__( *d __i__*) and π̂ __PS__( *t __j__*) can be derived in a relatively straightforward manner from two basic assumptions:

(1) all ( *d _i_, t _j_*) pairs of unknown activity are actually active, i.e. a__*__( *d _i_, t _j_*) ⇒ *a*_+_( *d _i_, t _j_*) = 1, for all a__*__( *d _i_, t _j_*) ∈ A_*_; and

(2) all ( *d _i_, t _j_*) pairs of unknown activity are actually inactive, i.e a__*__( *d _i_, t _j_*) ⇒ *a*_−_( *d _i_, t _j_*) = 1 for all a__*__( *d _i_, t _j_*) ∈ A_*_.

In the first case the magnitudes of ε̂ __PP__( *d __i__*) and ε̂ __PS__( *t __j__*) determine the respective uncertainties of π̂ __PP__( *d __i__*) and π̂ __PS__( *t __j__*), while in the second case, assuming that all ( *d _i_, t _j_*) pairs of unknown activity are in fact inactive gives values of π̂ __PP__( *d __i__*) and π̂ __PP__( *t __j__*) that are lower bounds to their true values. But as noted earlier their true values may be lower because of measurement, computational, or other types of errors.

The mathematical expressions in Equation (23) show that the true values, π __PP__( *d __i__*) and π __PS__( *t __j__*), are bounded, *i.e.*

$$\begin{array}{l} {\hat{\pi }}_{\text{PP}}(d_{i})\leq \pi_{\text{PP}}(d_{i})\leq {\hat{\pi }}_{\text{PP}}(d_{i})+{\hat{\varepsilon }}_{\text{PP}}(d_{i})\\ {\hat{\pi }}_{\text{PS}}(t_{j})\leq \pi_{\text{PS}}(t_{j})\leq {\hat{\pi }}_{\text{PS}}(t_{j})+{\hat{\varepsilon }}_{\text{PS}}(t_{j}) \end{array}\;(23)$$

and thus they depend directly on the magnitudes of their corresponding uncertainties, ε̂ __PP__( *d __i__*) and ε̂ __PS__( *t __j__*). Maximum upper bounds to these quantities are given by max[π̂ __PP__( *d __i__*)] = | T | and max[π̂ __PS__( *t __j__*)] = | D |, since the maximum connectivity of any *d __i__* node is equal to the total number of *t __j__* nodes, | T |, and similarly the maximum connectivity of any *t __j__* node is equal to the total number of *d __i__* nodes, | D |. If all of the *d* nodes are connected to all of the *t* nodes the network is fully connected, and thus would be a complete bipartite network. This result is clearly seen in Figure 2 if all of the edges were colored green and in Equation (13) if all of the matrix elements *a*_+_( *d _i_, t _j_*) = 1, a situation that is only achieved in the case where there are no inactive or unknown elements, i.e. *a*_( *d _i_, t _j_*) = a__*__( *d _i_, t _j_*) = 0 for all and *d __i__* ∈ D and *t __j__* ∈ T. Lastly, consider the case where all of the edges correspond to active or inactive drug-target pairs, *i.e.* there are no drug-target pairs of unknown activity. In this case, all of the edges in the network are either green or red, and the elements of the three adjacency matrices satisfy *a*_+_( *d _i_, t _j_*) + *a*_−_( *d _i_, t _j_*) = 1 and a__*__( *d _i_, t _j_*) = 0 for all *d __i__* ∈ D and *t __j__* ∈ T.

Applying the expressions in Equation (23) to the data in Table 2 and Table 4 yields the bounds given in Table 5 and Table 6. As discussed earlier, these bounds are unrealistically small, since in real cases the sizes of ε̂ __PP__( *d __i__*) and ε̂ __PS__( *t __j__*) are likely to be much larger than those used in the simple example presented here. Nevertheless, it illustrates a number of relevant points. In carrying out this analysis it is important to remember that all drug-target pairs whose activity has not been determined must be included in the class of drug-target pairs of unknown activity, which directly contributes to the uncertainty in π̂ __PP__( *d*) and π̂ __PS__( *t*).

**Table 5.** Upper and lower bounds to the degree of polypharmacology for the set of eight drugs in the simple example described in this work.

	Lower	Upper
***d*_1_**	3	4
***d*_2_**	2	3
***d*_3_**	1	3
***d*_4_**	3	3
***d*_5_**	2	3
***d*_6_**	2	2
***d*_7_**	4	4
***d*_8_**	2	3

**Table 6.** Upper and lower bounds to the degree of polyspecificity for the set of four targets in the simple example described in this work.

	Lower	Upper
***t*_1_**	5	7
***t*_2_**	5	6
***t*_3_**	4	7
***t*_4_**	5	5

## Summary and conclusions

The study of polypharmacology is becoming increasingly important in drug research because it raises awareness of the inherent lack of specificity of drugs and xenobiotics for specific targets. Moreover, it provides a basis for understanding the prevalence of side effects and the rationale behind the repurposing of drugs for new therapeutic indications. The concept of polyspecificity, on the other hand, affords support for the lack of specificity of drug targets. A simple mathematical argument shows that these seemingly disparate characteristics of drugs and targets are, in fact, closely related, a result that to the best of our knowledge has not been previously published by other authors. This is supported by a growing number of structural studies that suggest that the variety of different structural patterns arising in drug-target interactions is so large it is highly unlikely that high degrees of specificity in these interactions will occur.

Constructing networks is a popular enterprise in biology nowadays. Although useful, these networks have some significant limitations. For example, while they offer a highly visual depiction of the interrelationships among entities associated with the nodes in the network it is difficult to extract detailed information from them when the number of entities is large, a situation that also obtains in the case of drug-target networks. The issue can be overcome by utilizing the adjacency matrix of the network, which provides a faithful representation of its edge structure, and thus preserves the relations associated with active drug-target pairs. Because of this the degrees of polypharmacology and polyspecificity can be computed directly from adjacency matrices.

There is other information associated with drug-target pairs that is rarely if ever dealt with. Representing this information involves the use of the edge-colored bipartite drug-target networks introduced in this paper. In addition to representing active drug-target pairs, which is the case with standard drug-target networks, these augmented networks represent data associated with inactive drug-target pairs and with pairs of unknown activity. By including this heretofore latent data it is possible to compute global and local measures of data completeness as well as bounds for the degrees of polypharmacology and polyspecificity. These parameters can be viewed as diagnostics of the suitability of a given analysis of a drug-target network.

In the simple example describe here, the values for the uncertainties ε̂ __PP__( *d*) and ε̂ __PS__( *t*) are quite small, and hence the upper bounds lie close to the values of π̂ __PP__( *d*) and π̂ __PS__( *t*). This is not likely to be the case in larger, more realistic drug-target networks. In such cases, the uncertainties will be considerably larger due to a lack of data availability. As noted above, the reliability of the analysis can be increased by the use of experimentally or computationally determined data on inactive drug-target pairs. Unfortunately, such data is not as readily available in many publicly accessible databases where the focus is largely on drugs that are active with respect to specific targets. Assuming drugs without activity data are inactive, as is the case in the use of ‘decoys’ to test various computational methodologies, clearly leads to a loss of information. This trend needs to be reversed.

Although the analysis presented here is useful, it is just a start and by no means exhausts the possibilities for further study. Three areas for to consider for future research include:

(1) Expanding statistical analysis of drug-target network properties;

(2) Examining higher-order drug-target interactions; and

(3) Developing weighted and fuzzy representations of drug-target networks.

A lot of work is still needed in order to provide a suitably rigorous formalism for treating drug-target networks in ways that allow maximum extraction of information, which clarifies a number of the subtle issues associated with these biologically important networks.
