# Supplementary material for: A simple mathematical approach to the analysis of polypharmacology and polyspecificity data
Source: F1000Res. 2017 Jun 6;6:Chem Inf Sci-788. [Version 1] doi: 10.12688/f1000research.11517.1 (PMC5482344; doi:10.12688/f1000research.11517.1)
Supplement: Peer review history

# Peer review: Referee response for version 1

**DOI:** 10.5256/f1000research.12441.r23321

**Published:** 2017-06-20

**Authors:** Tudor I. Oprea

**Affiliations:** 1 Translational Informatics Division, Department of Internal Medicine, University of New Mexico School of Medicine, Albuquerque, NM, USA

This subject is relevant for the drug discovery community. The theoretical approach is potentially sound, as far as I can tell - but (full disclosure) while familiar with statistics and matrices algebra, I believe someone more competent in such mathematics should judge that part of the publication.

 The problem is **genuine**: Indeed, after more than a century of pharmaceutical research, it has become clear (owing to high throughput screening of large chemical libraries) that *many drugs bind to multiple targets. *This problem is compounded by other aspects such as **tissue distribution**, **on- and off- dissociation constants**, **half-life** and other pharmacokinetics parameters. Target and drug (*see below) specific elements influence the relevance of both polypharmacology and polyspecificity.

 Which begs the question, *how relevant is target polyspecificity?* The authors encode *"structurally dissimilar drugs"* in their definition (see Abstract and Introduction). This in itself is a slippery slope, considering Maggiora's 2006 Commentary that similar molecules do not always share the same activity landscape. The implication being that structural similarity does not always work. So, dis-similarity would have to be defined... at the 2D level (which fingerprints)? 3D? (shape? electrostatics? etc.). In my opinion, polyspecificity does NOT require "dissimilar" in the definition.

 Polyspecificity is relevant when one considers drugs co-administered simultaneously - with the possibility of exacerbating some side-effects or, perhaps, staying "on target". This is likely to occur, considering that 15% of U.S. adults are likely to use 5 or more prescription drugs (aka **polypharmacy**). Thus, the issue of target polyspecificity is relevant  and ought to be investigated more in the context of co-prescribed medications.

 The main topic of this paper is polypharmacology. The issue of **potency **appears to be brushed aside, as shown in the assumption that " *drug-target interactions can be characterized as binary relations*" (see Drug-Target Relationships). This, of course, implies that Drug D1, with a Ki of 1 nM (10 ^-9^ M) has the same relevance for polypharmacology and polyspecificity as Drug D2, with a Ki of 1 mM (10 ^-3^ M). In practice, this is not likely to be the case.

 Polypharmacology is not a binary issue of binding or not binding. The bi-partite drug-target network in Figure 1, therefore, not only has nodes and edges, but edges have values: D1 binds to target T1 with potency P1, D1 binds to T2 with P2 and so on... Which would change Table 2 into something more familiar to medicinal chemists, i.e., a Structure-Activity Table. 

 The issue of what's "active" vs. "inactive" (e.g., Fig 3) is a somewhat subjective issue. Take for example ropinirole: *"although the anti-Parkinsonian drug ropinirole is more potent at the D _3_ receptor than the D _2_ receptor by an order of magnitude, we annotate the D _2_ receptor as the mechanism of action target because D _2_ receptors, but not D _3_ receptors, are expressed in the substantia nigra, the pathologically relevant tissue for anti-Parkinsonian drugs"*. Our own DrugCentral entry shows other targets, such as the 5-HT _1A_ and alpha- _2B_ adrenergic receptors, with potency similar to D _2_ receptors. Is that relevant? Should all targets with potency below 6 (on the negative log scale) be considered "inactive"? The answer to these questions depends on the problem at hand.

 By the same token, the issue of polyspecificity may be regarded differently given a target for which over 20 potent (approved) drugs are known (some Receptor Tyrosine Kinases fit this profile), compared to a target for which only 2 drugs are approved (e.g., cyclin-dependent kinases 4 and 6).

 Given the wealth of data for drug-target interactions from a variety of sources such as ChEMBL, DrugBank, DrugCentral or GuideToPharmacology, it is recommended that real examples are used in this paper. Although " data completeness" remains an issue, the authors can no doubt identify a subset of 20-50 drugs, say anti-depressants or anti-psychotics, for which a wealth of *in vitro* bioactivity data are available through various channels, including PDSP in addition to the above.

 That would provide clear and immediate utility to the upper and lower bounds for the degree of polypharmacology (Table 5), which would make this paper more impactful. The authors are clearly aware of this, as discussed in Conclusions...

 I found the discussion related to the limitations of network biology representations particularly interesting. Perhaps that section could be expanded... 

(*) Footnote. Two simple scenarios are discussed. These do not include  target mutations (e.g., causing drug resistant cancers or infections), allelic variation, or other population-specific phenomena.  

1. The target is in the CNS, but the drug itself is an ABCB1 substrate (see for example the impact of ABCB1 on CNS side-effects), or the drug lacks blood-brain barrier permeability - in which case the potency of the drug *in vitro* is irrelevant *in vivo*. 
2. The drug can have significant *in vitro* potency on many targets, e.g., dobutamine hits over 20 human targets according to DrugCentral. However, its half-life is 2 minutes. Therefore, these "off target effects" are irrelevant.  

I have read this submission. I believe that I have an appropriate level of expertise to confirm that it is of an acceptable scientific standard, however I have significant reservations, as outlined above.

# Peer review: Referee response for version 1

**DOI:** 10.5256/f1000research.12441.r23318

**Published:** 2017-06-19

**Authors:** John Van Drie

**Affiliations:** 1 Van Drie Research LLC, North Andover, MA, USA

This is as close to 'publish as is' as I've ever seen. Excellent work, well articulated, good overview of literature.

 My only suggestion is that a paragraph at the end would be helpful, laying out the experimental implications of this theory, i.e. if this theory holds or if such analyses pan out, how would an experimentalist change their research plan?

I have read this submission. I believe that I have an appropriate level of expertise to confirm that it is of an acceptable scientific standard.

# Peer review: Referee response for version 1

**DOI:** 10.5256/f1000research.12441.r23316

**Published:** 2017-06-19

**Authors:** Karina Martinez-Mayorga

**Affiliations:** 1 Institute of Chemistry, National Autonomous University of Mexico, Mexico City, Mexico

This is an original and nice contribution to the field. The authors propose a mathematical approach to analyse the relation between polypharmacology and polyspecificity, that are, as presented here “two concepts running on the same avenue”. I particularly like the idea of extracting latent information to describe relationships between the degrees of these two complementary features.

 This work highlights the inherent complexity of biological systems providing a view of drug-target interactions as a pattern where both sides have an array of possibilities. Pattern recognition involved in the perception of odorants provides an additional example (See for instance DOI: 10.1038/81774) of the complexity involved in the recognition of ligands by biomacromoleules. It could be envisioned that the mathematical approach described in this paper will be attractive to parallel areas of biological processes governed by pattern interactions.

I have read this submission. I believe that I have an appropriate level of expertise to confirm that it is of an acceptable scientific standard.

## References

[1] AranedaRCKiniADFiresteinS: The molecular receptive range of an odorant receptor. *Nat Neurosci*.2000;3(12) : 10.1038/81774 1248-55 10.1038/81774 11100145

# Peer review: Referee response for version 1

**DOI:** 10.5256/f1000research.12441.r23317

**Published:** 2017-06-09

**Authors:** José L Medina-Franco

**Affiliations:** 1 DIFACQUIM Research Group, Department of Pharmacy, School of Chemistry, National Autonomous University of Mexico, Mexico City, Mexico

This well-written and organized manuscript addresses an extremely timely topic in drug discovery.

 The authors starts defining the basic concepts of polypharmacology and polyspecificity. Then, in a very clear and didactic manner (using nice illustrations), propose a general and intuitive mathematical approach to quantify the degrees of both concepts. It is clear from the manuscript the mathematical relationship of polypharmacology and polyspecificity (e.g., paraphrasing the authors “the two sides of the same coin”). The new measures address at some extent data incompleteness that is a major issue of chemogenomics data sets. As the authors point out in the Conclusions, this paper sets the ground to implement these metrics to public or private chemogenomics data sets. In particular, I found quite innovative and clear the edge-colored bipartite networks introduced in this manuscript.

 I strongly support indexing of this paper. Minor suggestions to further improve the manuscript:

  

1. The term “frequent hitter” related to polypharmacology can be added in the Introduction.
2. Comment on the effect of drug concentration in chemogenomics data sets. For instance, adverse drug reactions, and drug-interaction networks in general, will depend on the drug concentrations.
3. Page 4: Include reference related to the statement: “Recent work from Shoichet’s group at UCSF …”. I believe the authors refer to the paper published in ACS Chem. Biol. 2015 ^1^. This manuscript is not included in the Reference section of the current version.
4. Spell out "UCSF" (University of California at San Francisco).

I have read this submission. I believe that I have an appropriate level of expertise to confirm that it is of an acceptable scientific standard.

## References

[1] BarelierSSterlingTO'MearaMJShoichetBK: The Recognition of Identical Ligands by Unrelated Proteins. *ACS Chem Biol*.2015;10(12) : 10.1021/acschembio.5b00683 2772-84 10.1021/acschembio.5b00683 26421501PMC4684825
